# Palonosetron-Induced Ventricular Tachycardia in a Patient Receiving Cancer Chemotherapy

**DOI:** 10.7759/cureus.1480

**Published:** 2017-07-17

**Authors:** Prasad M, Shashidar V K, Ananya Chakraborty

**Affiliations:** 1 Department of Pharmacology, Vydehi Institute of Medical Sciences and Research Centre; 2 Vydehi Institute of Medical Sciences and Research Centre

**Keywords:** palonosetron, cinv, ventricular tachycardia

## Abstract

Chemotherapy-induced nausea and vomiting (CINV) is one of the major and most distressing adverse effects of cancer chemotherapy. It is treated with various antiemetic regimens, of which one class of drugs is 5-hydroxytryptamine type 3 receptor antagonists (5-HT_3_ RA). Palonosetron, a potent antiemetic, is a second generation 5-HT_3_ RA. All 5-HT_3_ antagonists, except palonosetron, have been reported to cause corrected QT interval (QTc) prolongation and certain arrhythmias. Here, we report a case of palonosetron-induced ventricular tachycardia in a 45-year-old patient receiving cancer chemotherapy.

## Introduction

Chemotherapy-induced nausea and vomiting (CINV) is a serious concern while treating patients for various cancers. CINV occurs in more than 90 percent of patients receiving highly emetogenic chemotherapy (HEC), 90 percent of patients receiving moderate emetogenic chemotherapy (MEC), and 10 to 30 percent and less than 10 percent in low and minimal emetogenic chemotherapy, respectively. It adversely affects the quality of life of patients, prolongs hospitalization, and increases health care costs [1].

CINV is treated with a number of currently available antiemetic agents. Some act better in the early phase, while others have better efficacy in the delayed phase of vomiting. Nowadays, one class of drugs that is widely used for CINV is 5-hydroxytryptamine type 3 receptor antagonists (5-HT3 RA). These drugs prevent emesis by blocking the binding of serotonin with 5-HT3 receptors on the nerve terminals of the vagus in the gastrointestinal tract. They also block the serotonin-mediated stimulation of the chemoreceptor trigger zone in the area postrema. They are more effective in controlling the delayed phase of CINV. The adverse reactions of 5-HT3 RA include headaches, dizziness, fatigue, diarrhea, and QTc prolongation [2].

Palonosetron is a second-generation 5-HT3 RA. It has a prolonged plasma half-life of around 40 hours. It has a strong binding affinity to the receptor that is almost 100 times higher than that of first-generation drugs. It also exhibits a specific allosteric, cooperative interaction with the receptor. It is a relatively safe, effective antiemetic with no clinically significant drug-drug interactions. It also has less adverse effects than other setrons. To date, there has been an absence of warning on the cardiac safety of palonosetron. However, here, we report a case of palonosetron-induced ventricular tachycardia in a 45-year-old patient receiving cancer chemotherapy.

## Case presentation

A 45-year-old male patient was admitted to the medical oncology department with complaints of a cough with expectoration. He also gave a history of finding it difficult to breathe since the last three months. His complaints were not associated with chest pain. He also complained of swelling in the neck and inguinal region since the last three months. The symptoms were insidious at the onset and gradually progressive in nature. There was no history of similar complaints in the family. The patient is a smoker and a nonalcoholic.

On general physical examination, he was found to have enlarged cervical, axillary, and inguinal lymph nodes. His systemic examination did not reveal any significant abnormalities. On investigation, his complete blood count, renal function test, and liver function tests were within normal limits. The two-dimensional echocardiography (2D Echo) showed normal valves and chambers with no regional wall motion abnormalities (ejection fraction (EF): 60 percent). A computerized tomography (CT) scan of the thorax revealed a nodular mass in the upper left lobe of the lung, with multiple mediastinal lymph nodes. The mass was 24x22x39 mm in size. There was evidence of a metabolically active upper lobe mass in the left lung with a few other lobe nodules, extensive lymphadenopathy (supra and infradiaphragmatic), and a left adrenal lesion likely representing lung carcinoma with metastasis, as revealed by a positron emission tomography-CT (PET-CT) scan. Liver, brain, and skeletal metastasis were absent. Initial immunohistochemistry (IHC) suggested squamous cell carcinoma.

The patient was started on injection (inj.) paclitaxel in the dose of 288 mg in 500 ml normal saline (NS) intravenous (IV) over three hours and inj. carboplatin in the dose of 750 mg in 500 ml 5 percent dextrose IV over one hour. He was administered inj. avil (pheniramine maleate), 22.75 mg IV stat; inj. rantac (ranitidine), 50 mg IV stat; and inj. dexamethasone, 8 mg IV stat. There were no significant adverse drug reactions.

However, in view of the significant lymphadenopathy, IHC was repeated and showed large cell neuroendocrine carcinoma of the lung. The relatives of the patient were counseled about the nature of the disease. The treatment was changed to a three-day course of six cycles of EP (etoposide + cisplatin). Each cycle was given after a three-week interval. Each cycle was started with a premedication of capsule aprepitant, 125 mg per oral stat on Day 1; 80 mg on Day 2 and Day 3; inj. palonosetron, 0.25 mg IV stat on Day 1; and inj. dexamethasone, 8 mg IV stat on all three days. 

**Figure 1:**
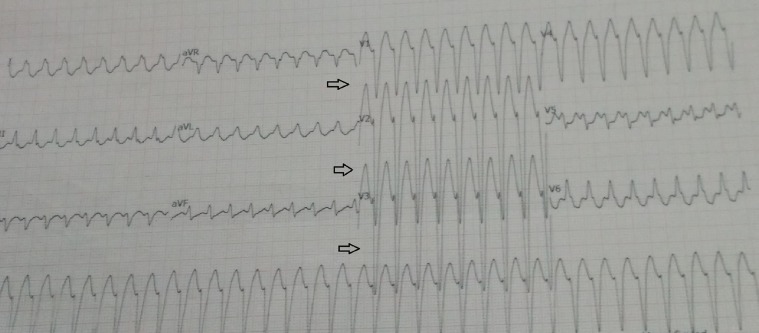
ECG tracing shows palonosetron-induced VT

The patient received EP#1 (inj. etoposide, 165 mg in 500 ml NS IV over three hours, and inj. cisplatin, 55 mg in 500 ml NS IV over three hours ) and EP#2 (inj. etoposide, 170 mg in 500 ml NS IV over three hours and inj. cisplatin, 56 mg in 500 ml NS IV over three hours) for three days, without any major adverse reactions. During the course of EP# 3 (inj. etoposide 170 mg and inj. cisplatin 56 mg), the patient complained of chest pain and shortness of breath after he was administered the same premedications. He was not started on chemotherapy medications but was immediately shifted to the emergency room for management. On examination, he was found to be conscious, with a heart rate of 208/min, blood pressure that was not recordable, and oxygen saturation (SPO2) that was 96 percent room air. Figure 1 is an electrocardiogram (ECG) trace showing ventricular tachycardia (VT). His pulse and blood pressure before starting the premedications were within normal limits

The patient was started on IV fluids, sedated with inj. midazolam, 2 mg IV stat. In view of the symptoms, a synchronized cardioversion with 150 J was performed. After all this, the patient reverted to a normal cardiac rhythm with a heart rate of 94/min. He improved symptomatically. He was later started on inj. amiodarone, 150 mg IV stat, as a preventive measure. He was shifted back to the ward, where there were no further episodes of VT. As a precautionary measure, subsequent cycles were continued without palonosetron.

A provisional diagnosis of palonosetron-induced VT was made. On Naranjo analysis, the adverse drug reaction (ADR) score was three, i.e., a probable reaction. 

## Discussion

Cancer chemotherapy is associated with a high incidence of nausea and vomiting, particularly with highly emetogenic chemotherapy (HEC) agents, such as cisplatin. Palonosetron is metabolized by multiple cytochromes P450 (CYP) enzymes, including CYP2D6, and, to a lesser extent, CYP3A and CYP1A2. The concomitant use of the inhibitors of these enzymes such as aprepitant, diltiazem, verapamil, amiodarone, and quinidine can increase the plasma concentration of the 5-HT3 antagonists and, subsequently, increase the risk for QT interval prolongation [3]. But, on a search of the literature, VT has not been reported earlier with the second-generation 5-HT_3_ RA, palonosetron.

Previous studies suggested that patients who are administered first-generation 5-HT3 RAs were at risk of experiencing adverse cardiac events, such as VT. This adverse cardiac event is predicted to be because of a blockade of cardiac potassium ion channels, resulting in QT prolongation. This, in turn, leads to VT. Previous meta-analysis ADR data on palonosetron reported constipation, headache, diarrhea, and dizziness as the most common effects [4].

A recent meta-analysis investigated the mean change in the corrected QT (QTc) interval in patients receiving palonosetron for CINV. QT interval prolongation was reported in three trials. The specific data evaluated in the reviewed studies showed that patients receiving palonosetron experienced a mean increase in the QTc interval after treatment, which was significantly lower than that with older antagonists [4]. However, there have been no reports of QTc leading to VT in previous studies.

Since palonosetron possesses a different structure and affinity to 5-HT3 receptors and cardiac ion channels, the drug might have unique effects on cardiac repolarization. Palonosetron causes less cardiac events within a therapeutic concentration as compared to older antagonists. It is effective against emesis within serum concentration levels not high enough for cardiac ion channel blockage [5].

## Conclusions

Recent pieces of evidence proved that palonosetron, a second generation 5-HT3 receptor antagonist, significantly adds to the clinician’s ability to effectively control CINV, particularly the delayed phase. The effective management of CINV improves the quality of the lives of patients undergoing cancer chemotherapy. With only a few reported cases of palonosetron-induced QTc changes, current labeling includes no warning regarding QT prolongation. However, physicians should be made aware that such an event can take place. They must prepare themselves to understand, diagnose, and treat such an emergency reaction with utmost care and expertise. 
